# Se-methylselenocysteine inhibits inflammatory response in an LPS-stimulated chicken HD11 macrophage-like cell model through the NFKB2 pathway

**DOI:** 10.3389/fvets.2024.1503436

**Published:** 2025-01-08

**Authors:** Min Yao, Binyu Wang, Zitong Li, Suqing Wu, Bingyu Zhao, Ning Sun, Huiping Xiao, Jianwu Wang, Guoping Liu, Tinghua Huang

**Affiliations:** ^1^College of Animal Science and Technology, Yangtze University, Jingzhou, China; ^2^College of Agriculture, Yangtze University, Jingzhou, China

**Keywords:** se-methyselenocysteine, inflammatory response, HD11, NFKB2, chicken

## Abstract

Among the various sources of selenium supplementations, the Se-methylselenocysteine (SeMC) is a natural organic selenium compound that has been demonstrated to have multiple advantages in terms of metabolism efficiency and biosafety in animals. Nevertheless, the genome-wide impact of SeMC on gene transcription remains to be elucidated. In this study, we employed an LPS-stimulated chicken HD11 macrophage-like cell model to identify the principal transcription factors involved in transcriptome regulation responsible for SeMC treatment. RNA-seq identified 3,263 transcripts that exhibited a statistically significant differential expression between the SeMC-treated group and the control group and 1,344 transcripts that exhibited a statistically significant differential expression between the LPS + SeMC- and LPS-treated groups (FDR < 0.05, FDR > 1.5). The bioinformatic analysis identified six transcription factors (NFKB2, RFX2, E2F5, ETV5, BACH1, and E2F7) as potential candidate genes for transcriptome regulation in SeMC-treated HD11 cells. Subsequent experimental verification demonstrated that SeMC suppressed the inflammatory response in an LPS-stimulated chicken HD11 cell model via the TXN2—NF-κB pathway. The administration SeMC was observed to reduce the production of ROS as well as the transcription and translation of inflammatory cytokines in both cell culture and *in vivo* animal studies. One candidate pathway by which SeMC exerts its effects is through the targeting of the transcription factor, NFKB2, by selenoprotein TXN2. This study identified key transcription factors and revealed one of the potential mechanisms through which SeMC exerts its anti-inflammatory effects from the perspective of transcriptional regulation.

## 1 Introduction

Selenium supplementation improves antioxidant capacity of cells in patients with coronary artery disease by increasing GPX-1 protein activity (1). Se-methylselenocysteine (SeMC) is a natural organic selenium source for humans and animals. It has higher bioavailability and is safer than inorganic selenium, such as sodium selenite (2). It has been demonstrated that 80% of the natural selenium found in allium species such as onion, leek, garlic, ramps, and brassica species including broccoli, radish, Brussels sprouts, cabbage, and milk vetch exists in the form of SeMC (3). Initially, SeMC was demonstrated to have cancer preventive activity (4). *In vitro* experiments have demonstrated that the anticancer mechanism of SeMC may be attributed to the induction of DNA fragmentation and apoptosis to the cancer cells (5, 6). In addition, SeMC may exert anticancer effects by inhibiting cell growth via PI3-K and its downstream effector molecules in tumor cells (7). Other studies demonstrated that SeMC enhanced the phosphorylation of AKT and glycogen synthase kinase 3β and protected cells from excessive oxidative stress by stimulating an antioxidant response (8). Moreover, the metabolism production of SeMC, namely, methylseleninic acid, has been demonstrated that can elevate REDD1 and inhibit prostate cancer cell growth (9, 10).

Recent studies have demonstrated that the SeMC has anti-inflammatory properties. SeMC has been demonstrated to reduce the nuclear translocation of the p65 and p50 subunits of nuclear factor-NF-κB (NF-κB) and diminish the phosphorylation of mitogen-activated protein kinases (MAPKs), including p38, MAPK, and c-Jun in a LPS-treated RAW 264.7 macrophage-like cell model (11). In addition, SeMC was observed to suppress the RNA levels of iNOS, TNF-*α*, IL-1β, IL-6, COX2, and MMP-9 in LPS-activated RAW 264.7 cells (12).

SeMC has been demonstrated to possess remarkable antioxidant, antitumor, and immunomodulatory properties. Nevertheless, there is a paucity of research examining the transcription factors that SeMC may potentially target. The aim of the present study is to identify transcription factors regulated by SeMC in an inflammatory cell model using RNA-seq and a newly developed bioinformatics tool, the Jinzer/Flaver suite (13, 14). The rationale of the study was to establish a correlation between the gene expression levels with transcription factor binding sites. Flaver software (13, 14), which implements weighted Kendall’s tau statistics, was employed to identify the principal transcription factors within each sample. The method implemented in Flaver allows for testing the significance of the correlation between the rank orders of genes within the gene set and their corresponding rank orders within the gene list. These findings will contribute to the further investigation of the anti-inflammatory properties of SeMC in chickens.

## 2 Materials and methods

### 2.1 Chemicals and reagents

The RPMI 1640 medium (11875085), fetal bovine serum (FBS, A3161001C), and penicillin–streptomycin (15140148) were purchased from Gibco™ (Gaithersburg, United States). The chicken serum (CS, S9080), protease inhibitor cocktail MIX (IKM1201), and phosphatase inhibitor cocktail MIX II (IKM1070) were purchased from Solarbio Life Science (Beijing, China). The MiniBEST Universal RNA Extraction Kit (9767), PrimeScript^™^ RT Reagent Kit (RR037A), and TB Green^®^ Premix Ex Taq^™^ II FAST qPCR kit (CN830A) were purchased from Takara (Beijing, China). The reactive oxygen species detection kit (S0033S), lipopolysaccharide (LPS) of *E. coli* serotype O55:B5 (ST1470), and protein A/G magnetic beads (P2179S) were bought from Beyotime Biotechnology (Shanghai, China). The enzyme-linked immunosorbent assay (ELISA) kits for interleukin-1β (IL-1β, J25734), interleukin-6 (IL-6, J25788), tumor necrosis factor-*α* (TNFα, J25759), and cyclooxygenase-2 (COX2, J26986) were purchased from Giled Biotechnology (Wuhan, China). The anti-NFKB2/p52 rabbit antibody (113157) was obtained from NovoPro (Shanghai, China). The SeMC (26046-90-2) was purchased from LKT Laboratories, Inc. The shRNA for NFKB2 was designed using the BLOCK-iT™ RNAi Designer from Thermo Fisher (5′-GCTGTGATCAAGCAGCTAATT-3′).

### 2.2 Cell culture

The chicken HD11 macrophage-like cells were kindly provided by Dr. Jiao Song (College of Life Science, Yangtze University). The HD11 cells were cultured in RPMI 1640 complete medium (containing 8% of FBS, 3% of CS, and 1% of penicillin–streptomycin), kept at 41°C in 5% CO_2_, and passaged every 3 days at a ratio of 1:3. Approximately 12 h before challenging, HD11 cells were seeded into 6-well cell plates at a concentration of 2 × 10^6^ cells per well in RPMI 1640 containing 100 μM SeMC, and the concentration of SeMC has been verified to be fit for cell viability (Supplementary Figure S1). The cells were stimulated with 100 ng/ mL lipopolysaccharide for 6 h, washed, and further lysed with 1 mL TRIzol reagent or 200 μL IP lysis buffer. The samples for RNA-seq experiment and pull-down mass spectrometry analysis were collected from four treatment groups with three replicates for each group, namely, the SeMC+PBS, SeMC+LPS, LPS, and Control (PBS). Samples for the assessment of reactive oxygen species (ROS) assays were collected from seven treatment groups, namely, the SeMC, LPS, SeMC+LPS, LPS+shRNA, SeMC+shRNA, SeMC+LPS+shRNA, and Control (PBS). The shRNA lentivirus for NFKB2 was transfected to HD11 cells in accordance with the manufacturer’s instructions.

### 2.3 Animals and sample collection

The animal experiments conducted in this study were performed in accordance with the Regulations for the Administration of Experimental Animals, as issued by the Science and Technology Commission of China (NO. 2006-398). Furthermore, all procedures involving animals were approved by the Animal Ethics Commission of Yangtze University (Jingzhou, Hubei, China). A total of 80 healthy 1-day-old white leghorn chickens (40 male and 40 female) with similar body weights were managed with the following method. The relative humidity was consistently maintained at 40–60%. The temperature within the feeding room was maintained at 32–34°C, with weekly reductions of 2°C until it reached the final range of 22–24°C. The facility was cleaned out every day, maintained in a hygienic condition, with waste products removed daily, and the air supply in the house was kept fresh. The experimental period spanned 21 days, during which the chickens were fed a standard chicken diet (in accordance with the NY/T33-2004 standard, as outlined in Supplementary Table S1) from day 1 to day 7. On the seventh day, the chickens were randomly divided into four groups with 20 replicates (10 male and 10 female). The first group (Control) was fed with a standard diet, the second group (LPS) was fed with a standard diet plus injection of 1 mg/kg body weight LPS intraperitoneally at day 21st, the third group (SeMC) was fed with a standard diet containing 0.3 mg/kg SeMC, and the last group (SeMC + LPS) was fed with a standard diet containing 0.3 mg/kg SeMC, plus an injection of 1 mg/kg of body weight of LPS intraperitoneally on day 21st. After challenging for 12 h, 6 mL of blood was harvested from the wing vein of the birds. A total of 3 mL of the collected blood samples were injected into an anticoagulant tube containing ethylenediaminetetraacetic acid for RNA extract. An addition of 3 mL of blood samples could be permitted to coagulate, after which the serum was separated by centrifugation at 4°C and 2,000× g for 10 min. It was then stored in a refrigerator at −80°C for further ELISA.

### 2.4 High-throughput sequencing and identification of differentially expressed genes

The cells were lysed with 1 mL TRIzol reagent and subsequently snap-frozen in liquid nitrogen. They were then sent to the DNA facility with dry ice for RNA-seq. The sequencing libraries were prepared using the Illumina TruSeq RNA Sample Preparation Kit, following the manufacturer’s instructions (Illumina Inc., United States). Approximately 10 μg of total RNA from each sample was used for library construction and RNA sequencing. The sequencing was conducted on an Illumina HiSeq 2500 (Illumina Inc., United States) using the single-read sequencing protocol. The data were filtered to obtain clean reads and remove low-quality reads present in the raw reads, in accordance with the recommendations of the manufacturer. Hisat2 (15) was employed to map the clean reads to the reference genome (GRCg7b), which was extracted from the NCBI genome database (16). The estimated read reads count per gene was calculated using HTSeq (17) and used for the comparison of gene expression differences among samples. The limma R package (18) was employed for the calculation, and the criteria for differentially expressed genes were set as a false discovery rate (FDR) < 0.05 and a fold change (FC) > 1.5. All data discussed in this study have been deposited in the NCBI GEO database (19) under the accession number GSE278197 (https://www.ncbi.nlm.nih.gov/geo/query/acc.cgi?acc=GSE278197).

### 2.5 The identification of the key transcription factors playing roles in the transcriptome

The FC and FDR values for genes detected by RNA-seq were converted into RANK values (raw score of differential expression, RDE) in the ranked gene list using Equation 1 as recommended by Flaver’s method (13) which was specified by Formula (1). The FC represented the fold change of gene, and FDR summarized the false discovery rate of genes between samples. A total of 1,575 position weight matrices (PWMs) for TFs were collected from Jaspar (20) and HOCOMOCO (21) databases. The chicken genome sequence was obtained from the NCBI’s FTP site. Run Jinzer software (14) with the PWMs and the promoter sequence (550 bp) identified a total of 4.52 million TF binding site (TFBS) with Jindex score ≥ 1.0 (a higher Jindex score indicated higher possibility to be true TFBS). The gene set was constructed by associating target genes with transcription factors if the gene was identified to possess at least one TFBS for the TF. A rank value was assigned to each TF-(target gene) pair, calculated by the Jindex score of the Jinzer’s output.


(1) $$RDE=-sgn\left(\log_{2}FC\right)\;\cdot \;2^{\mathrm{abs}\left(\log_{2}FC\right)}\;\cdot \;\log_{10}FDR$$

The RDE values of all genes were used to create the RNAK gene list. We used the Flaver software (13) which implements the weighted Kendall’s tau statistics, for the identification of the key transcription factors playing roles within each tissue. The method applied in this study tested the significance of correlation between the rank orders for genes in the gene set and their corresponding rank orders within the gene list. As illustrated by Shieh, the relationships between the gene set and the gene list, in terms of agreement in the ranks, in gene set enrichment analysis can be measured by a weighted rank correlation (22). Let Si and Li, *i* = 1, …, *n* be the ranks of the gene set and gene list, respectively. Furthermore, let (*i*, *R_i_*), *i* = 1, …, *n*, be paired ranks, where *R_i_* is a rank entity of *L* whose corresponding *S* has rank *i* among *Sj*, *j* = 1, …, *n*. As discovered by Shieh, the weighted Kendall’s tau has the form of Equation 2 (22).

(2) $$\tau_{w}=2/\left[{\left(\overset{n}{\underset{i}{\sum }}v_{i}\right)}^{2}-\overset{n}{\underset{i}{\sum }}v_{i}^{2}\right]\;\cdot \;\overset{n}{\underset{i>j}{\sum }}v_{i}v_{j}sgn\left(i-j\right)sgn\left(R_{i}-R_{j}\right)$$

The sgn(x) = −1, 0 or 1, if *x* <, = or > 0. The vi represents the weighting function which is bound to [1, *n*] and range in (0, 1). The limiting distribution (LD) can be derived as Equation 3. When *n* → ∞, LD approximated to *N*(0, 1) and the *p*-values can be estimated (22).

(3) $$LD=\sqrt{n}\tau_{w}\frac{3\lim_{n\to \infty }n^{-1}\sum_{i}^{n}v_{i}}{2\sqrt{\lim_{n\to \infty }n^{-1}\sum_{i}^{n}v_{i}^{2}}}$$

The weighting function v_i_ used in this study is the mixed density of RNAK value of the gene set and the gene list, range for 0 to 1 (specified by –w 7 option in Flaver), to address a questions, that is, see how the genes in top ranked TF-(target gene) pairs in the gene set correlated with the top ranked genes in the gene list. Other parameters of Flaver were setting to the default.

### 2.6 RNA isolation and real-time PCR assay

The total RNA was extracted from chicken blood samples using the TRIzol method. The isolated RNA was reverse-transcribed into cDNA using a PrimeScript^™^ RT reagent Kit following standard protocols. The PCR primers are listed in Supplementary Table S2. Real-time PCR was conducted using a TB Green^®^ Premix Ex Taq^™^ II FAST qPCR on a BIORAD Cycler. Gene expression was quantified using the 2^−ΔΔCT^ method, with the data being normalized to the expression of GAPDH.

### 2.7 Pull-down mass spectrum analysis

The 2 × 10^6^ of cell pellets were washed three times using a wash buffer containing 0.1% Tween 20, 150 mM NaCl, 50 mM HEPES, and 2 mM MgCl_2_ at 10,000 g for 1 min at room temperature. The cells were then lysed by the addition of 200 μL of IP lysis buffer (comprising 1.0% NP-40, 0.2 mM EDTA, 20 mM pH 8.0 Tris–HCl, 180 mM NaCl, 1% of a 100× phosphatase inhibitor cocktail, and 100× protease inhibitor cocktail). After centrifugation at 12,000 rpm for 10 min at 4°C, the supernatants were incubated with anti-NFKB2 p52 rabbit polyclonal antibody and protein A/G magnetic beads at 4°C overnight. Subsequently, the samples were separated by a magnetic separator and denatured by SDS loading buffer, after which they were examined by matrix-assisted laser desorption/ionization time-of-flight mass spectrometry (MALDI-TOF MS).

### 2.8 Enzyme-linked immunosorbent assay

The levels of TNF-*α*, IL-1β, IL-6, and COX2 in serum were quantified using ELISA kits specified in the chemicals and reagents’ section for chickens after the necessary dilution according to the manufacturer’s instructions. The ELISA experiments were conducted on three samples collected from independent birds for each group.

### 2.9 Flow cytometry experimental analysis

The intracellular ROS were quantified using the 2′,7′-dichlorodihydrofluorescein diacetate (DCFHDA) assay. In brief, 1 mL of DCFHDA (10 μM) was added to each well of a 6-well plate with a cell layer, in which the cell culture supernatant had been removed. The plate was then incubated at 37°C for 30 min. The cells were rinsed three times with serum-free cell culture medium. The flow cytometry data were obtained using a BD FACS Melody cell sorter cytometer. All density plots are representative of three independent experiments, with 8,000–20,000 events acquired for all experiments. The data were analyzed using FlowJo 10.4. The production of ROS was calculated as the ratio of DCFH-DA^+^ cells within the gates.

## 3 Results

### 3.1 SeMC regulates the transcriptome of chicken HD11 macrophage-like cells

**Figure 1:**
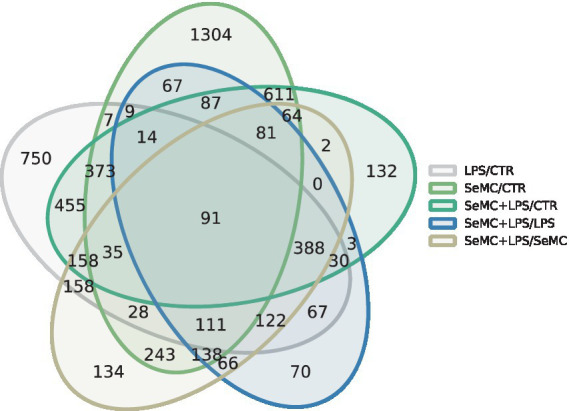
Numbers of differentially expressed genes in SeMC-treated HD11 macrophage-like cells. A summary of the differentially expressed transcripts in response to SeMC treatment (LPS/CTR, SeMC/CTR, SeMC+LPS/CTR, SeMC+LPS/LPS, SeMC+LPS/SeMC) is presented in Venn diagrams. The identification of these genes was conducted using a linear mixed model, with the FDR (false discovery rate) controlled at less than 0.05 and FC (fold change) higher than 1.5.

We performed RNA-seq on a total of 12 samples, and an average of 48 million sequences per sample was obtained. These sequences were mapped to 16,162 transcripts, with an average sequencing depth of approximately 327×. The statistic results revealed that 3,263 transcripts exhibited significantly differential expressions (FDR < 0.05 and FC > 1.5) between the SeMC-treated group and the control (CTR) group. In the LPS treatment model, SeMC treatment induced a significantly differential expression of 1,344 transcripts (SeMC+LPS/LPS, FDR < 0.05 and FC > 1.5). In the SeMC treatment model, LPS can result in the significant differential expression of 1,819 transcripts (SeMC+LPS/SeMC, FDR < 0.05 and FC > 1.5). The Venn diagram illustrating the number of differentially expressed genes among the groups is presented in Figure 1. The detailed gene lists for each group comparison are shown in Supplementary Data S1. In the SeMC+LPS/LPS comparison group, the most significantly differentially expressed transcripts were C1QB (upregulated by 9.73-fold), BTN3A3L2 (downregulated by 1.96-fold), NFKB2 (upregulated by 3.18-fold), PMEPA1 (upregulated by 2.64-fold), C1QC (upregulated by 7.12-fold), and C1QTNF9B (downregulated by 1.74-fold). The annotation shows that 20 genes in the significantly differentially expressed gene list were associated with the innate immune response, including JCHAIN, EXFABP, TLR2A, CATHB1, OASL, HCK, and ANXA1. Furthermore, 16 genes are directly associated with the defense response to bacteria, including CEBPB, EXFABP, CHGA, IRF8, IL8L2, NOS2, COCH, AVBD3, LYZ, AVBD6, and MPEG1.

### 3.2 The identification of transcription factors involved in SeMC regulation

**Figure 2:**
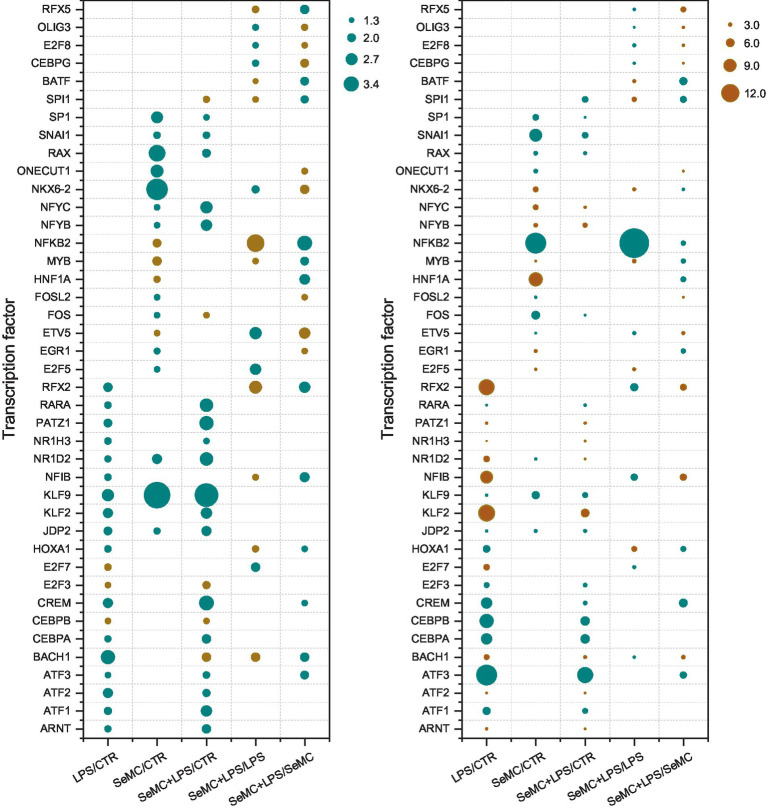
Transcription factors identified by Flaver in the SeMC-treated HD11 macrophage-like cells. The plot on the left illustrated the transcription factors of which Jindex value of their target genes was significantly correlated with the RDE value determined by the RNA-seq experiment. The positive correlation transcription factors were colored in blue, and negative correlation transcription factors were colored brown. The right plot depicts the expression level of the transcription factors, upregulated transcription factors were colored by blue, and downregulated transcription factors were colored by brown. The size of the bubble in both the left and right plot indicates the level of significance which is calculated by −log_10_(score).

The Flaver analysis revealed that 16 transcription factors were involved in the regulation of SeMC+LPS/LPS differentially expressed genes, and 20 transcription factors were involved in the regulation of SeMC+LPS/SeMC differentially expressed genes. The Jindex values of NFKB2, RFX2, BACH1, HOXA1, MYB, SPI1, NFIB, and RFX5 exhibited a negative correlation with the RDE values of SeMC+LPS/LPS and displayed a positive correlation with the RDE values of SeMC+LPS/SeMC. The Jindex values of E2F5, ETV5, E2F7, NKX6-2, CEBPG, E2F8, MEF2C, and OLIG3 were found to be positively correlated with the RDE values of SeMC+LPS/LPS and negatively correlated with the RDE values of SeMC+LPS/SeMC. HOXA1, NFKB2, E2F5, MYB, NKX6-2, and SPI1 exhibited decreased expression in the SeMC+LPS/LPS group. The expressions of BACH1, E2F7, NFIB, RFX2, ETV5, CEBPG, E2F8, MEF2C, OLIG3, and RFX5 were increased in SeMC+LPS/LPS and decreased in the SeMC+LPS/SeMC group. The results are shown in a bubble plot in Figure 2.

**Figure 3:**
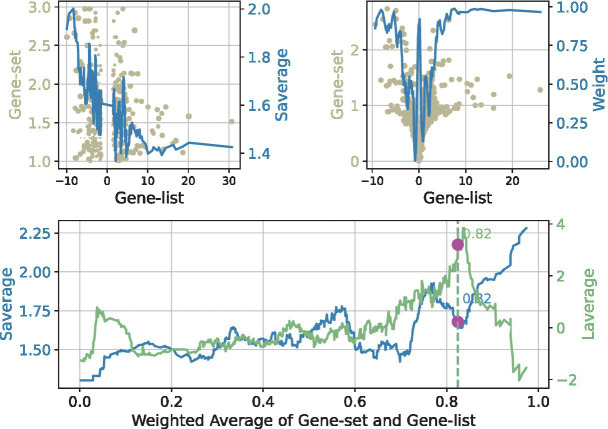
Visualization of the Flaver’s result, take the NFKB2 gene set in SeMC+LPS/LPS gene list as an example. The results of the Flaver’s analysis were plotted by first sorting the genes by RDE (top-left panel) or weighted RANKs (lower panel) and then using a sliding window with a size of 0.02n to traverse from the highest value to the lowest value. The distribution of Jindex (light blue) and RDE (light yellow) in the window was then calculated. The term “Saverage” is used to denote the weighted value of gene set values, whereas “Laverage” represents the weighted values of gene list.

The results of Flaver’s statistic for NFKB2, a representative transcription factor identified in this study, are presented in Figure 3. First, the data were sorted by Jindex, and thereafter, a sliding window with a size of 0.02n was used to slide from the highest value of RDE to the lowest value, and the distribution of Jindex in the window was calculated. Subsequently, a sliding window was employed to traverse from the highest value of weighted RANKs to the lowest value, with the distribution of RDE and Jindex within the windows calculated. In the upper region of the window, the Jindex values of the genes tend to be distributed toward the upper end, but the RDE values tend to be distributed toward the lower end. This suggested that genes with higher Jindex values tend to be downregulated. The forementioned analysis results indicate that NFKB2 plays a negative transcriptional regulatory role in this experiment, which is consistent with the results reported in the literature that NFKB2 is a transcriptional suppressor (23).

### 3.3 The identification of TXN2 as a selenium protein partner of NFKB2

**Figure 4:**
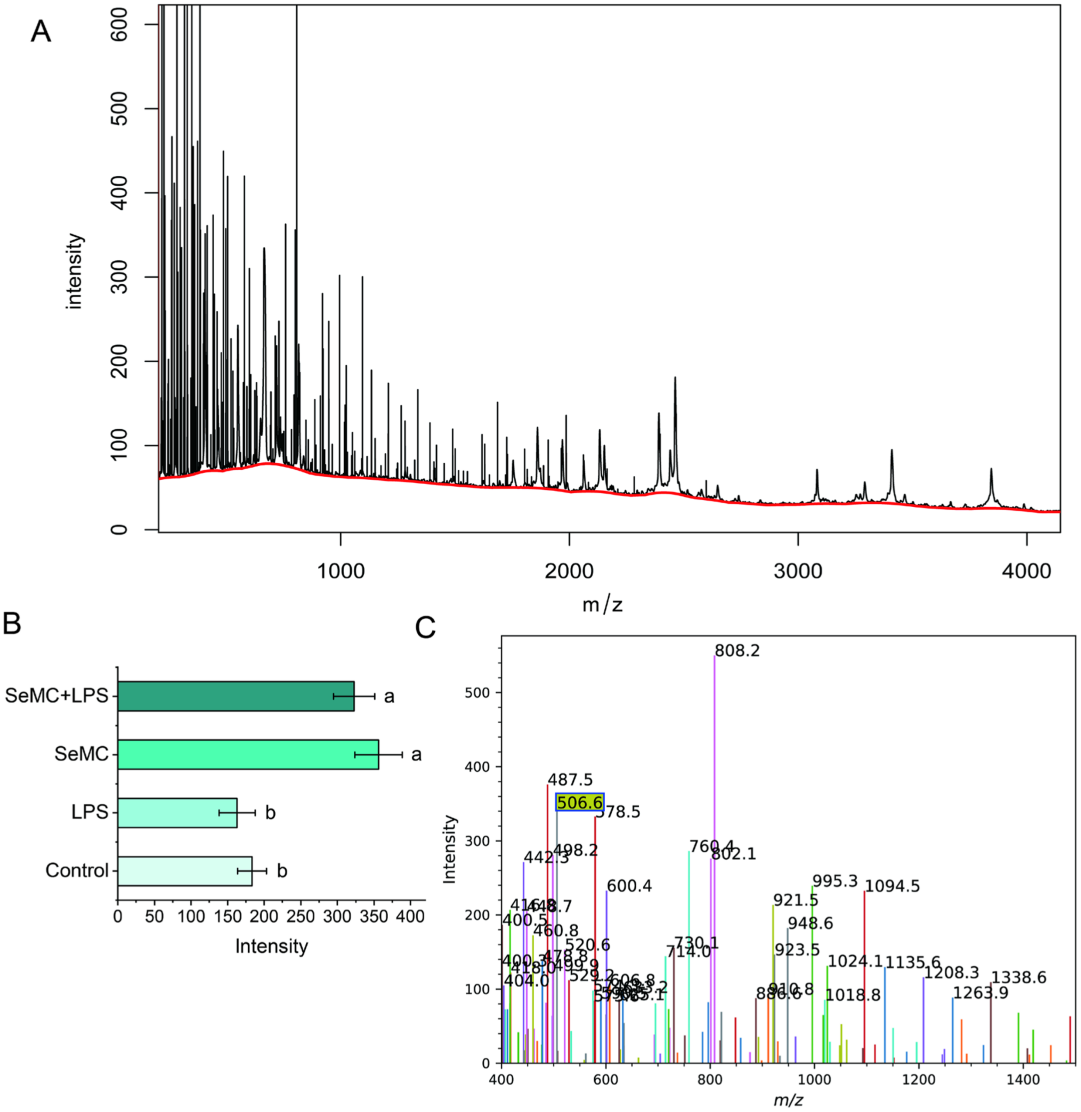
Protein levels of selenium protein TXN2 in LPS-treated HD11 macrophage-like cells analyzed by MALDI-TOF mass spectrometry. The raw data were presented in plot **(A)**. The characteristic peak (at m/z of 506.6) represents an ion fragment dissociated from the 16.50 kDd polypeptide containing the selenocysteine residue at the 151st amino acid was shown in plot **(C)**. The levels of the selenium protein (intensity of the selenocysteine residue containing ion fragments) were illustrated in bar graph in plot **(B)**. Bars bearing different letters are significantly different from each other (*p* < 0.05).

According to the BIOGRID database (24), the selenium proteins that can interact with the NF-κB complex include TXN2, MIEN1, ATP1A1, MIEN1, and AIFM1. TXN2 can be translated into two polypeptides. The longer polypeptide contains a selenocysteine residue at the 151st amino acid, with a size of approximately 16.50 kD, while the shorter polypeptide terminates at the 150th codon (AUG), with a size of approximately 16.26 kD. MALDI-TOF mass spectrum analysis demonstrated that HD11 cells cultured in SeMC medium had a distinctive peak at m/z 506.6, indicating the “LIGAUS” ion fragment (Figures 4A,C). This ion fragment is dissociated from the 16.50 kD longer polypeptide which contains the selenocysteine residue at the 151st amino acid. The expression of TNX2 was found to be significantly upregulated in HD11 cells that has been co-cultured with a medium containing SeMC (Figure 4B, *p* < 0.05). The quantity of peptide chains containing selenocysteine residues increased significantly when treated with SeMC, with a difference of approximately 2-fold for both the SeMC- and SeMC+LPS-treated groups.

### 3.4 The expression of transcription factors in peripheral blood collected from birds administrated with SeMC and LPS

**Figure 5:**
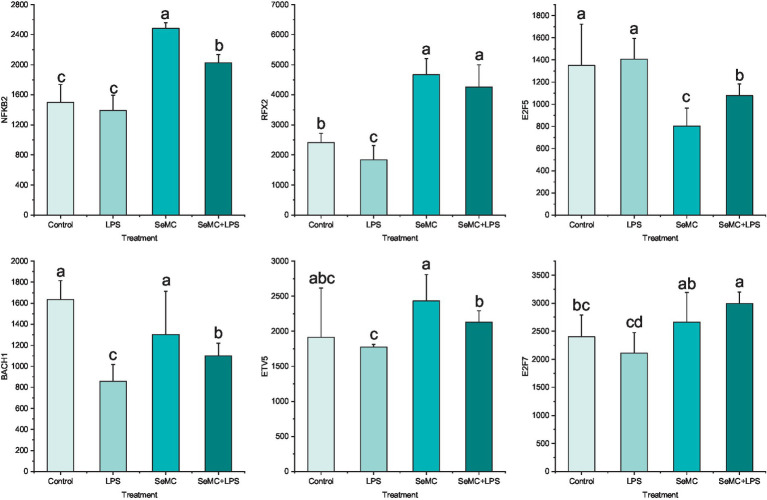
Real-time PCR validation of the transcription levels of six selected transcription factors in peripheral blood collected from chickens administered with SeMC and LPS. The height of the bars represents the relative transcriptional levels of the genes. Bars bearing different letters indicate a significantly different from each other (*p* < 0.05).

The expression levels of NFKB2, RFX2, E2F5, ETV5, BACH1, and E2F7 in peripheral blood collected from chickens administered with SeMC and LPS were analyzed using real-time fluorescence quantitative PCR (Figure 5). The results demonstrated that the expression levels of NFKB2, RFX2, BACH1, ETV5, and E2F7 in the SeMC+LPS treatment group were significantly higher than those in the LPS treatment group. The expression levels of NFKB2, BACH1, and ETV5 in the SeMC+LPS treatment group were significantly lower than those in the SeMC treatment group. The expression levels of E2F5 in the SeMC+LPS treatment group were significantly lower than those in the LPS treatment group, while the expression levels in the SeMC+LPS treatment group were significantly higher than those in the SeMC treatment group. These results were generally consistent with those of the RNA-seq experiment.

### 3.5 The analysis of the downstream factors of NFKB2 in SeMC and LPS administrated chickens

**Figure 6:**
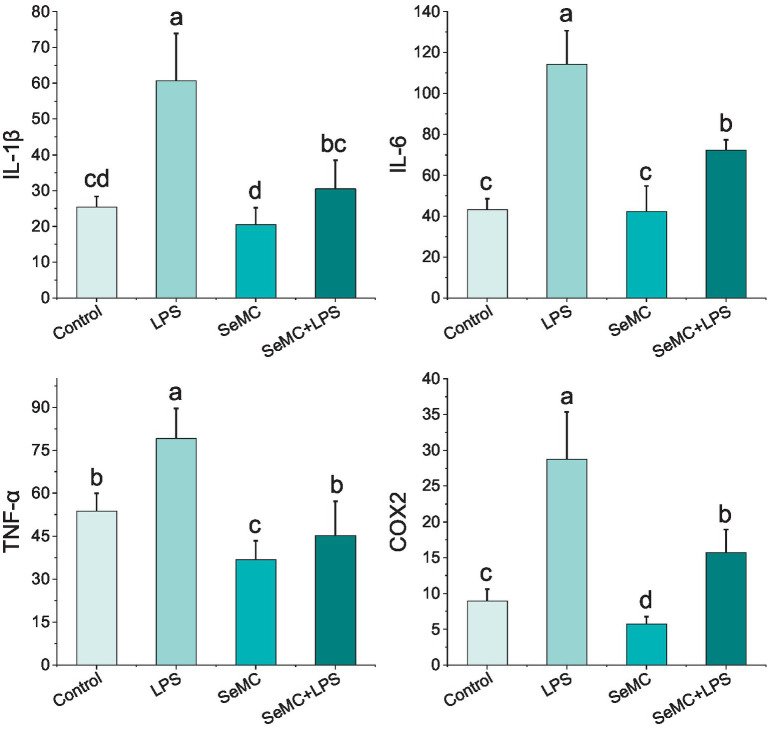
Analysis of protein levels of four NF-κB downstream inflammation factors in peripheral blood of SeMC or LPS administrated chickens by ELISA. Bars bearing different letters indicate a significantly different from each other (*p* < 0.05).

We employed the ELISA method to detect four NF-κB downstream factors in the peripheral blood collected from chickens administrated with SeMC and LPS, including IL-1β, IL-6, TNF-α, and COX2. The results demonstrated that the IL-1β levels in chickens treated with SeMC and SeMC+LPS markedly reduced in comparison with those in chickens treated with LPS (*p* < 0.05). The IL-6 levels in chickens treated with SeMC+LPS and SeMC were significantly lower than those treated with LPS (*p* < 0.05). However, no significant difference was observed between chickens treated with SeMC and those treated with PBS alone (Control). The TNF-α and COX2 levels in chickens treated with SeMC and SeMC+LPS were significantly lower than in chickens treated with LPS (*p* < 0.05) (Figure 6).

### 3.6 A shRNA targeting NFKB2 reverted the ROS levels in SeMC in HD11 macrophage-like cells

**Figure 7:**
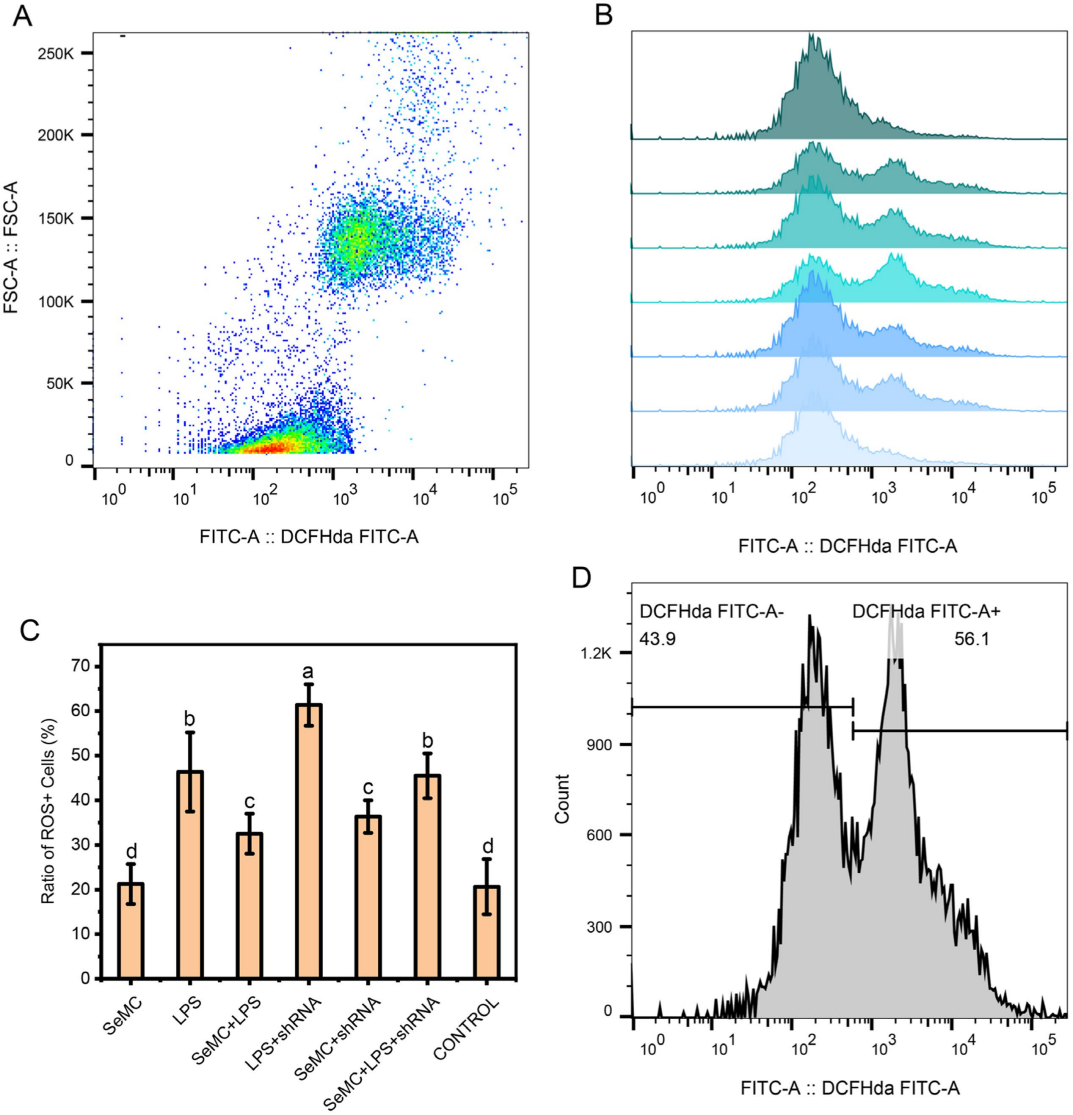
ROS levels for SeMC- and LPS-treated HD11 macrophage-like cell samples. The flow cytometry results for the CONTROL, LPS+shRNA, LPS, SeMC+LPS+shRNA, SeMC+LPS, SeMC+shRNA, and SeMC samples are presented in a histogram in plot **(B)**, from top to bottom. The average ratios of ROS^+^ cells for three replicated samples are presented in bar graph in plot **(C)**. The scatter plot and gate information are shown in plots **(A,D)**, respectively.

Treating HD11 cells with SeMC, LPS and shRNA targeting NFKB2 demonstrated that the LPS + shRNA group exhibited the highest ROS levels (*p* < 0.05), ranking as the first group. The ROS levels of LPS and SeMC+LPS + shRNA were found to be the second group, and the SeMC+LPS and SeMC+shRNA ranked as the third group. The ROS levels of the second group were found to be significantly lower than the first group (*p* < 0.05). The ROS levels of SeMC and Control group were significantly lower than those of the first three groups (*p* < 0.05). The results are presented in Figure 7, and the raw data are available in Supplementary Data S2. In addition, over-expression of TXN2 in LPS-stimulated HD11 cells cultured within SeMC-containing medium using a TXN2 caring vector resulted in a significant downregulation of the expression of IL-1β (2.31-fold), IL-6 (2.52-fold), TNF-*α* (1.85-fold), and COX2 (3.23-fold) with a *p* < 0.05.

## 4 Discussion

The administration of organic including selenocystine and selenomethionine had been demonstrated to induce dose-dependent apoptosis in A375, HepG2, and MCF7 cell lines, accompanied by a notable elevation in intracellular ROS levels (25–27). Furthermore, DNA strand breaks were also observed in organic selenium-treated HepG2 and MCF7 cell lines (25). The inhibition of ROS can effectively reduce the oxidative damage of cells triggered by L-selenocysteine (28). Nevertheless, it is possible that MeSC may exert a distinct regulatory influence on the generation of ROS comparing to other organic selenium sources. MeSC has been demonstrated to induce to introduce a transient increase and subsequent decrease in cellular reactive oxygen species, which appears to be associated with the elevation of nuclear factor erythroid 2-related factor 2 (NRF2), a pivotal transcription factor for the antioxidant response, that can protect cells from excessive oxidative stress (8). It has proposed that SeMC may inhibit oxidative stress and inflammation subsequent to nerve injury by upregulating the Nrf2/HO-1 pathway (1). SeMC nanoparticles (NPs) have been demonstrated to protect the viabilities and functions of cultured hepatocytes in drug- or chemical-induced acute hepatotoxicity models via effective removal of ROS (29).

In this study, NFKB2, RFX2, ETV5, BACH1, and two E2F transcription factors (E2F5 and E2F7) have been identified as candidate genes involved in regulatory process of ROS. Among these six factors, NFKB2 has been widely known to act as an inhibitor of NF-κB (23). The rank values of the Jindex of NFKB2’s target genes’ TFBS exhibited a negative correlation with the rank values of gene differential expression levels in the SeMC+LPS/LPS group. In addition, NFKB2 was significantly upregulated in the SeMC+LPS/LPS group, indicating that, when NFKB2 was highly expressed, the target genes with higher binding scores tended to be lowly expressed. The aforementioned analysis demonstrated that NFKB2 played an pivotal role in transcriptional regulatory regulation within the LPS-stimulated chicken HD11 macrophage-like cell model treated with SeMC. The regulation effect on its target genes was negative, indicating that selenocysteine may exert an anti-inflammatory effect by activating NFKB2 pathway.

Approximately 20 kinds of selenoproteins have been identified in animals, and the majority of these selenoproteins exhibit enzymatic redox function through selenocysteine (30–32). Glutathione peroxidase (GSH-Px) is one of the most important selenocysteine-containing enzymes, which catalyzes the reduction of H_2_O_2_ and other peroxides, thereby protecting cells from free radicals and reactive oxygen species (ROS). Thioredoxin reductase plays a regulatory role in its metabolic activity by catalyzing the reduction of thioredoxin and may play an important role in the prevention and treatment of cancer (33). Thyroid hormone deiodinase is a crucial link between selenocysteine and thyroid hormone metabolism, which is vital for normal fetal growth and development (32). The result of this experiment demonstrated that the expression of selenoprotein TXN2 was significantly upregulated after SeMC treatment. Furthermore, TXN2 has been shown to bind to NF-κB complex, which lends support to the existing literature reporting that TXN2 can regulate the function of NF-κB (34, 35). The interaction of TXN2 with the NF-κB complex has the potential to impair its activity, thereby reducing the inflammatory response.

**Figure 8:**
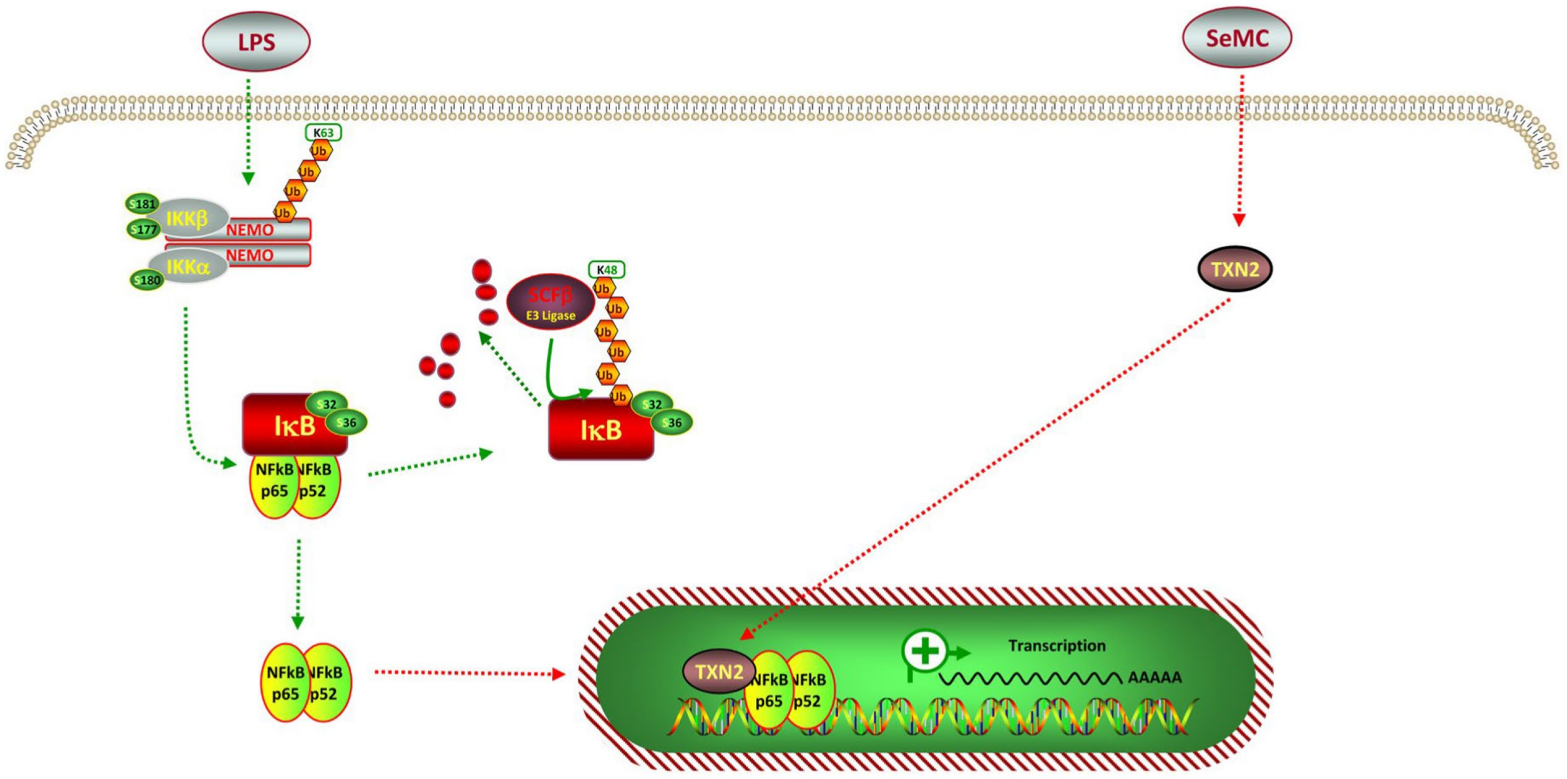
Pathway for Se-methylselenocysteine inhibits intermediated inflation in a LPS-stimulated chicken HD11 macrophage-like cell model. The p52 is a analog of the amino-terminal half of p100, which is encoded by NFKB2. The p52 protein is devoid of the transcription activation domains and can function as a repressor of NF-κB-specific transcription. The p65 protein is encoded by the RELA gene, containing both DNA-binding domains and the transcription activation domains. These domains can server as transcription activation domain of the NF-κB complex.

It is widely accepted that SeMC was is associated with oxidation (25–27), and the NF-κB pathway is known to regulate the generation of ROS (36–38). It is noteworthy that the expression of NFKB2-regulatedinflammatory cytokines, including TNF-*α*, IL-1β, IL-6, and COX2, was markedly diminished following SeMC treatment. In addition, SeMC treatment resulted in a reduction in intracellular ROS levels, which could be reverted by the inhibition of the NFKB2 gene. This suggested that SeMC treatment may attenuate the inflammatory response through the NFKB2 pathway. One potential intermediate effect molecule is the selenoprotein TXN2, which binds to the RELA subunit (p65) of NF-κB complex (Figure 8). This binding let enables TXN2 to act as an analog for NFKB2, thereby suppressing the activity of the NF-κB complex. These findings elucidated one potential mechanism through which SeMC exerts anti-inflammatory effects, from the vantage point of transcriptional regulation. The results provided a useful pathway for targeting inflammatory responses of chicken stimulated by LPS.

## Data Availability

The datasets presented in this study can be found in online repositories. The names of the repository/repositories and accession number(s) can be found in the article/Supplementary material.
