# Supplementary material for: Se-methylselenocysteine inhibits inflammatory response in an LPS-stimulated chicken HD11 macrophage-like cell model through the NFKB2 pathway
Source: Front Vet Sci. 2025 Jan 8;11:1503436. doi: 10.3389/fvets.2024.1503436 (PMC11751066; doi:10.3389/fvets.2024.1503436)
Supplement: Supplementary file 4 [file Data_Sheet_4.DOCX]

Supplemental document

Supplemental Fig. 1 Cell viability of HD11 cells treated with different concentrations of SeMC and LPS.

Supplemental Table 1. Formular of standard chicken diet

| Ingredient | Content (%) |
| --- | --- |
| Corn | 56.0 |
| Soybean meal | 32.5 |
| Corn gluten meal | 3.4 |
| Soybean oil | 3.0 |
| Limestone | 1.2 |
| Dicalcium phosphate | 2.1 |
| L-Lysine | 0.4 |
| DL-Methionine | 0.1 |
| Sodium chloride | 0.3 |
| Premix | 1.0 |
| Total | 100 |
| Calculated nutrient levels |  |
| Apparent metabolizable engery, MJ/kg | 12.76 |
| Crude protein, % | 21.36 |
| Calcium, % | 0.97 |
| Total phosphorus, % | 0.67 |
| Available phosphorus, % | 0.47 |
| Lysine, % | 1.23 |
| Methionine, % | 0.49 |
| Methionine + cystine, % | 0.83 |
| Analyzed nutrient levels |  |
| Crude protein, % | 20.81 |
| Calcium, % | 1.11 |
| Total Phosphorus, % | 0.71 |
| Lysine, % | 1.26 |
| Methionine, % | 0.50 |

Premix provided per kilogram of diet: vitamin A (transretinyl acetate), 10,000 IU; vitamin D3 (cholecalciferol), 3,000 IU; vitamin E (all-rac-α-tocopherol), 30 IU; menadione, 1.2 mg; Thiamin, 2.1 mg; riboflavin, 6 mg; nicotinamide, 40 mg; choline chloride, 400 mg; calcium pantothenate, 10 mg; pyridoxine·HCl, biotin, 0.04 mg; folic acid, 1 mg; vitamin B12 (cobalamin), 0.013 mg; Fe (from ferrous sulfate), 80 mg; Cu (from copper sulphate), 6.0 mg; Mn (from manganese sulphate), 100 mg; Zn (from zinc oxide), 50 mg; I (from calcium iodate), 1.1 mg; Se (from sodium selenite), 0.2 mg.

Supplemental Table 2. Sequence of primers for Real-time PCR

| Gene symbol | Forward primer | Revise primer |
| --- | --- | --- |
| NFKB2 | ACATGACGGCACAGTTCAGC | CTGAGCCCGCAGTCTTGTCC |
| RFX2 | GGCCTCGTGTACATTTGCAT | TACACAGCTCCAGGCATCTCG |
| E2F5 | GCTTTACTCCCTTTGGACGTT | CAGCACGAGTCATCAACCTG |
| ETV5 | CCACCTCTCATATATACGTCCCT | ACTGAGCAAATGTTCCTAGCCAT |
| BACH1 | CGCCTCATGGACTTAATTCCG | TATGCAGGCTCCAAGCGTTC |
| E2F7 | CGTTGCTGTTGACATCACGTT | CCTCATAAGGCTGTTATTCGTT |
